# *In vivo* bioassay to test the pathogenicity of missense human *AIP* variants

**DOI:** 10.1136/jmedgenet-2017-105191

**Published:** 2018-04-09

**Authors:** Elena Daniela Aflorei, Benjamin Klapholz, Chenghao Chen, Serban Radian, Anca Neluta Dragu, Nina Moderau, Chrisostomos Prodromou, Paulo S Ribeiro, Ralf Stanewsky, Márta Korbonits

**Affiliations:** 1 Centre for Endocrinology, Barts and the London School of Medicine, Queen Mary University of London, London, UK; 2 Department of Physiology, Development and Neuroscience, University of Cambridge, Cambridge, UK; 3 Department of Cell and Developmental Biology, Division of Biosciences, Faculty of Life Sciences, University College London, London, UK; 4 Department of Endocrinology, C.I. Parhon National Institute of Endocrinology, Carol Davila University of Medicine and Pharmacy, Bucharest, Romania; 5 Protein Dynamics and Cell Signalling Laboratory, Centre for Tumour Biology, Barts Cancer Institute, Queen Mary University of London, London, UK; 6 Genome Damage and Stability Centre, University of Sussex, Brighton, UK; 7 Institute of Neuro- and Behavioural Biology, Westfälische Wilhelms University, Münster, Germany

**Keywords:** AIP, drosophila melanogaster, FIPA, pathogenic genetic variant, pituitary adenoma

## Abstract

**Background:**

Heterozygous germline loss-of-function mutations in the aryl hydrocarbon receptor-interacting protein gene (*AIP*) predispose to childhood-onset pituitary tumours. The pathogenicity of missense variants may pose difficulties for genetic counselling and family follow-up.

**Objective:**

To develop an *in viv*o system to test the pathogenicity of human *AIP* mutations using the fruit fly *Drosophila melanogaster*.

**Methods:**

We generated a null mutant of the *Drosophila AIP* orthologue, *CG1847,* a gene located on the Xchromosome, which displayed lethality at larval stage in hemizygous knockout male mutants (*CG1847^exon1_3^*). We tested human missense variants of ‘unknown significance’, with ‘pathogenic’ variants as positive control.

**Results:**

We found that human *AIP* can functionally substitute for *CG1847*, as heterologous overexpression of human *AIP* rescued male *CG1847^exon1_3^* lethality, while a truncated version of *AIP* did not restore viability. Flies harbouring patient-specific missense *AIP* variants (p.C238Y, p.I13N, p.W73R and p.G272D) failed to rescue *CG1847^exon1_3^* mutants, while seven variants (p.R16H, p.Q164R, p.E293V, p.A299V, p.R304Q, p.R314W and p.R325Q) showed rescue, supporting a non-pathogenic role for these latter variants corresponding to prevalence and clinical data.

**Conclusion:**

Our *in vivo* model represents a valuable tool to characterise putative disease-causing human *AIP* variants and assist the genetic counselling and management of families carrying *AIP* variants.

## Introduction

Pituitary adenomas arise from hormone-secreting cells of the anterior pituitary gland. The presenting symptoms are due to either excess or deficiency of pituitary hormones or local space-occupying effects. Loss-of-function mutations in the aryl hydrocarbon receptor-interacting protein gene (*AIP*) predispose to an autosomal dominant disorder with incomplete penetrance (20%–23%) associated usually with growth hormone-secreting pituitary adenomas leading to acromegaly or gigantism.^1–3^ To date, more than 100 different *AIP* variants have been identified, with the majority (75%) resulting in a missing or truncated AIP protein.^2 3^ A change in amino acid sequence due to missense variants could affect protein folding and stability^4^ and may alter the availability of protein–protein interaction sites. The C-terminus of AIP includes conserved tetratricopeptide repeat (TPR) domains, and alterations in key amino acids are known to disrupt secondary structure, leading to unstable proteins.^5–7^ While pathogenicity is beyond doubt for the truncating mutations, establishment of pathogenicity for missense variants can be challenging, posing therefore a key question for clinical genetic counselling and decision making.^8^

The strategies employed to establish pathogenicity for heterozygous tumour suppressor genes, such as *AIP*, include: allele frequency in the general population, loss of the wild-type (wt) allele in the tumour (loss of heterozygosity (LOH)), in silico prediction pipelines,^9^ *in vitro* functional studies and evaluation of variant segregation with the phenotype in large pedigrees.^10^ LOH analysis of tumourous tissue has also been exploited to determine the pathogenic role of *AIP* variants,^11 12^ and AIP immunostaining is significantly reduced in the majority but not in all patients carrying *AIP* mutations.^13–15^ *In vitro* functional studies have also been employed to evaluate the protein stability of *AIP* variants,^4^ their effect on cell proliferation^13^ and their interaction with PDE4A5^13 16^ and RET,^17^ but these assessments are necessarily indirect.

However, the *in vivo* consequences of *AIP* missense variants have never been investigated. We aimed to develop an *in vivo* strategy to help determine the pathogenicity of missense *AIP* variants.

## Materials and methods

### Fly stocks and genetics

The *Drosophila melanogaster* strains used in this study: *w^iso^* (gift from Nic Tapon, London, UK), *yw;Bl/CyO (*Lindsley and Zimm),^18^ w* P{EP}CG1847^G1839^ (Bloomington Drosophila Stock Center: Stock ID: 32600),^19^ *yw;; Ki, pp, Δ2–3, P{CaryP}attP40* embryos (BestGene Inc, California, USA) and *yw; Act-Gal4/CyO.*

### *Drosophila* husbandry

Fly crosses were maintained at 25°C. For counting, the rescued males crosses were flipped every 9–10 days to prevent the mix of individual flies from different generations.

### Generation of mutant CG1847 flies: imprecise excision screen

The *CG1847* gene was mutated by P-element transposase-mediated deletion of genomic DNA. For this, a fly line was obtained, in which a P-element is inserted within the 5′UTR of *CG1847*: w*P{EP}CG1847^G1839^ (Bloomington *Drosophila* Stock Center).^20^ Females homozygous for the *CG1847* mutation are not viable, while heterozygous mutant females develop normally. The resulting stocks were screened by PCR, and the putative mutants were identified via Sanger sequencing. Sequence chromatograms were visualised and analysed using the BioEdit Sequence Alignment Editor software (http://www.mbio.ncsu.edu/bioedit/bioedit.html) (Ibis Biosciences, Carlsbad, California, USA).

### Rescue of *CG1847* function

A genomic rescue construct containing the regulatory and coding regions of *CG1847* (2763 bp) was generated, cloned into the pW@RpA vector (kindly provided by Professor Nick Brown’s laboratory, Cambridge, UK, details available on request).

To obtain the genomic rescue construct for hAIPwt, the AIP cDNA insert (1001 bp) was amplified from a pcDNA3-Myc-AIPwt vector.^13^ To obtain the genomic rescue construct of truncated *AIP*, the last 86 bp encoding for the seventh alpha helix were deleted. Transgenic lines for 11 different h*AIP* mutations (p.I13N, p.R16H, p.W73R, p.Q164R, p.C238Y, p.G272D, p.E293V p.A299V, p.R304Q, p.R314W and p.R325Q) were also generated. Mutagenic primers were designed using the Stratagene’s QuickChange Primer Design program at www.stratagene.com/qcprimerdesign. The QuickChange XL Site-Directed Mutagenesis kit (Agilent Technologies) was used, and mutagenesis was done according to standard recommended procedure.

All transgenic lines were generated by injecting the rescue constructs into *Drosophila y^1^w^67c23^; P{CaryP}attP40* embryos, which enabled the generation of transgenic stocks with constructs on chromosome 2. These transgenic fruit flies stocks were balanced over the balancer chromosome *CyO.* For males resulting from the rescue crosses, the h*AIP* transgene (online supplementary figure 4B,C: middle panels) was detected using primers against human *AIP* cDNA. In addition, the presence of Y chromosome (bottom panels) was detected using a set of primers for the *Ppr-Y* gene.

10.1136/jmedgenet-2017-105191.supp1 Supplementary file 1

### Statistical analysis

Experimental data sets were analysed in JMP (SAS institute). Statistical comparisons were analysed with one-way analysis of variance followed by a Tukey-Kramer test. Data are presented as mean ±SEM. A value of P<0.05 was considered to be statistically significant.

### Western blotting analyses

The different UAS (Upstream Activation Sequence) insertions for the human *AIP* were confirmed to drive protein expression in combination with the *actin-GAL4* using specific commercially available antibody. The Western blots were incubated overnight, at 4°C, with primary antibody anti-AIP/ARA9 Mouse Monoclonal^21^ (Novus Biologicals) at a dilution of 1:1000. Anti-Beta Tubulin, Mouse monoclonal (E7 Developmental Studies Hybridoma Bank)^22^ was used as a loading control at a dilution of 1:15 000. Secondary antibody IRDye 680 LT Goat anti-Mouse IgM (LI-COR Biotechnology) was used at a concentration of 1:1000. Odyssey Infrared Imaging System (LI-COR) was used for image acquisition. Results are representative of four independent western blot analyses from two independent experimental replicates.

## Results

### Characterisation of the *Drosophila* orthologue of human *AIP*

The *D. melanogaster* gene *CG1847* (NM_132530.4)^23^ is the fruit fly’s single orthologue of human *AIP.* This three-exon gene is located on chromosome X at position 10F2, base pair (bp) 11 869 170 to 11 871 168 (Drosophila genome release August 2014). As it is located on the X chromosome, male flies will have only one copy of this gene.

**Figure 1:**
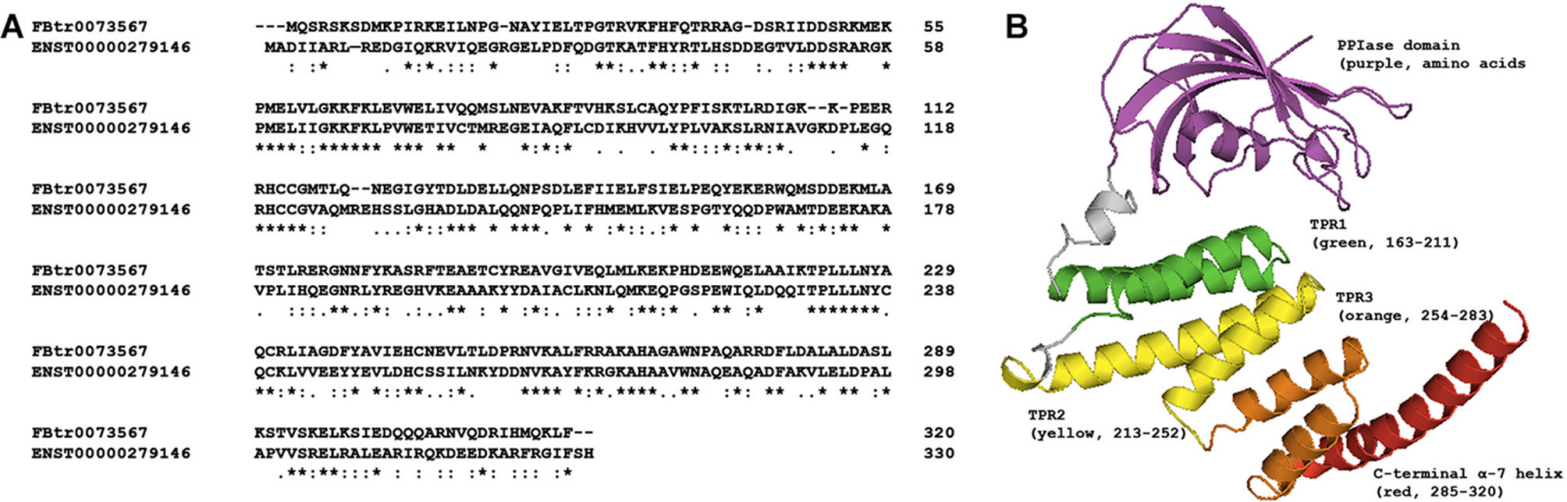
Comparison of *Drosophila* CG1847 and human AIP proteins. (A) The human and *Drosophila* proteins are similar; they share 120 identical amino acids, 80 strongly conserved and 34 weakly conserved amino acids. Stars indicate identity, and colons and dots indicate high and low similarity amino-acids, respectively. (B) A three-dimensional predicted model of CG1847 indicates that the *Drosophila* protein has a similar structure to its human orthologue, with an N-terminal PPIase domain, three pairs of conserved antiparallel alpha-helices forming the tetratricopeptide repeat domains (TPRs) and the final extended α-helix, α−7. AIP, aryl hydrocarbon receptor-interacting protein gene.

*CG1847* is a previously uncharacterised *Drosophila gene*, and no phenotypic fruit fly data are available in public databases. Protein alignment of CG1847 (FBtr0073567) and human AIP (hAIP, ENST00000279146) was performed by Clustal Omega^24^ and revealed a shared overall identity of 38% (Figure 1A). The three-dimensional theoretical model of CG1847 (Figure 1B) revealed a protein structure that closely resembles the published AIP protein structure, with a typical N-terminal peptidyl-prolyl *cis-trans* isomerases (PPIase)-like domain^25^ and the C-terminal TPR domains.^26^

### *CG1847* is an essential gene and its loss results in early lethality

**Figure 2:**
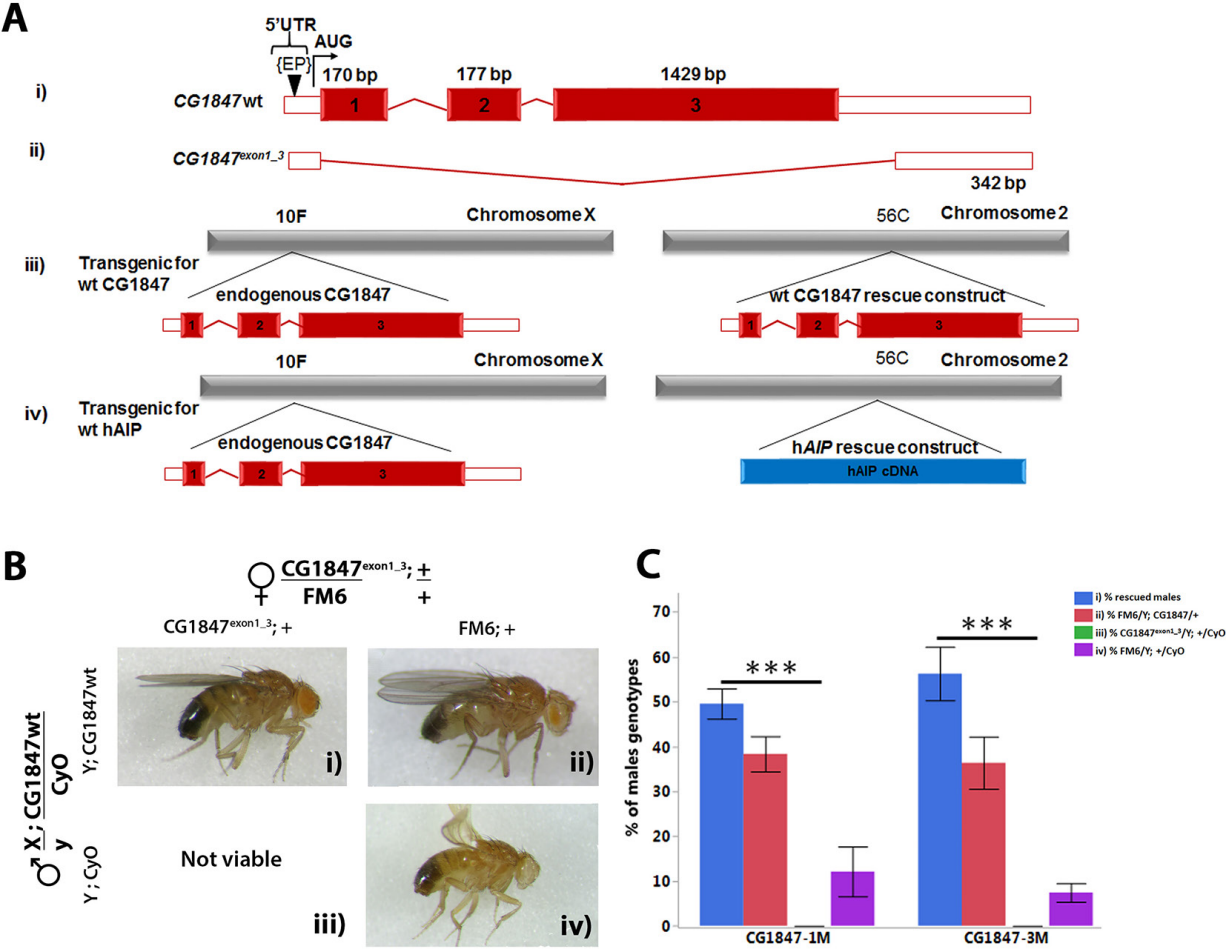
The lethality of *CG1847* mutants can be rescued by expression of wild-type (wt) *CG1847* under the control of its own promoter. (A) Schematic diagram of the wt *CG1847* (i), *CG1847^exon1^* ^_3^ (ii), *CG1847* rescue construct and (iii) transgenic generated human *AIP* rescue construct (iv). The diagram represent the mRNA, with red boxes representing coding region and white boxes representing 5′ and 3′ untranslated regions. (ii) The *CG1847* mutant was generated by imprecise P-element excision of P-CG1847^G1839^ (EP in the figure). (iii) Schematic representation of transgenic animals with genomic rescue construct containing the regulatory and coding regions of *CG1847* inserted on the second chromosome. (iv) Schematic representation of transgenic animals with the *AIP* cDNA rescue construct inserted on the second chromosome. (B) Transgenic males carrying the *CG1847* genomic rescue construct and balanced over CyO were crossed to the *CG1847^exon1_3^* heterozygous females and examined for their ability to rescue male lethality. Segregation of alleles and the possible combinations are shown in the lateral panels. Panel (i): male carrying the mutant *CG1847* allele inherited from their mothers rescued by the wt *CG1847* allele on the second chromosome. Panel (iii): males are not viable as they carry the *CG1847* mutant allele and lack the genomic rescue construct from the paternal chromosome 2. Panels (ii) and (iv) depict male progeny lacking the *CG1847* mutation. (C) Statistical analysis of rescue experiments with the *CG1847* genomic rescue construct (n=4). The associated letters (panels i–iv) correspond to the phenotypes depicted in Figure 2B. X-axis labels *CG1847-1M* and *CG1847-3M* represent two different transgenic stocks carrying the rescue construct. Error bars represent SE of the mean. Asterisks indicate statistical significance as determined by Student’s t-test (**P<0.01). AIP, aryl hydrocarbon receptor-interacting protein gene; bp, base pairs.

We used imprecise P-element excision^27^ to generate a loss-of-function mutations in *CG1847* (Figure 2A). One of the resulting lines harboured an excision of 2511 bp covering all three exons (*CG1847^exon1_3^*, Figure 2A-ii). The deletion did not extend into the neighbouring genes *CG2025* and *CG11802*, positioned 279 bp and 129 bp downstream of the *CG1847* 5′ and 3′ UTR regions, respectively. *CG1847* is located on the X chromosome, and no viable hemizygous males were observed carrying this mutation. Because heterozygous females were normal, viable and fertile, this suggests that complete loss of *CG1847* leads to lethality, similarly to mouse knockout models.^28 29^

To demonstrate that lethality of *CG1847^exon1_3^* is solely due to the deletion of *CG1847* coding sequence and not caused by additional mutations generated by the imprecise excision, transgenic flies carrying a genomic rescue construct on chromosome 2, containing the wt regulatory and coding regions of *CG1847* (Figure 2A-iii), were generated and injected into *Drosophila* embryos (BestGene). Transgenic male flies were crossed with heterozygous *CG1847^exon1_3^* mutant females (Figure 2B) to study the ability of the wt *CG1847* rescue construct to reverse the lethality of *CG1847^exon1_3^* males. Hemizygous mutant *CG1847^exon1_3^* males expressing the wt *CG1847* construct on chromosome 2 were viable (Figure 2B, panel i), suggesting that lethality of *CG1847^exon1_3^* flies is indeed due to loss of *CG1847*. The degree of rescue was determined by analysing the proportion of each viable male genotype in the second generation (Figure 2C). All offspring without endogenous *CG1847* or without the rescue construct (Figure 2B, panel iii) died at the larval stage. However, a small number of males without the rescue construct, but phenotypically similar to males in Figure 2B, panel iii, were viable due to a meiotic non-disjunction event in the previous generation, a low-frequency phenomenon common in *Drosophila* genetics (online supplementary material 1, figures 1–3). 30–33

### Human *AIP* is able to functionally compensate for *CG1847* loss

**Figure 3:**
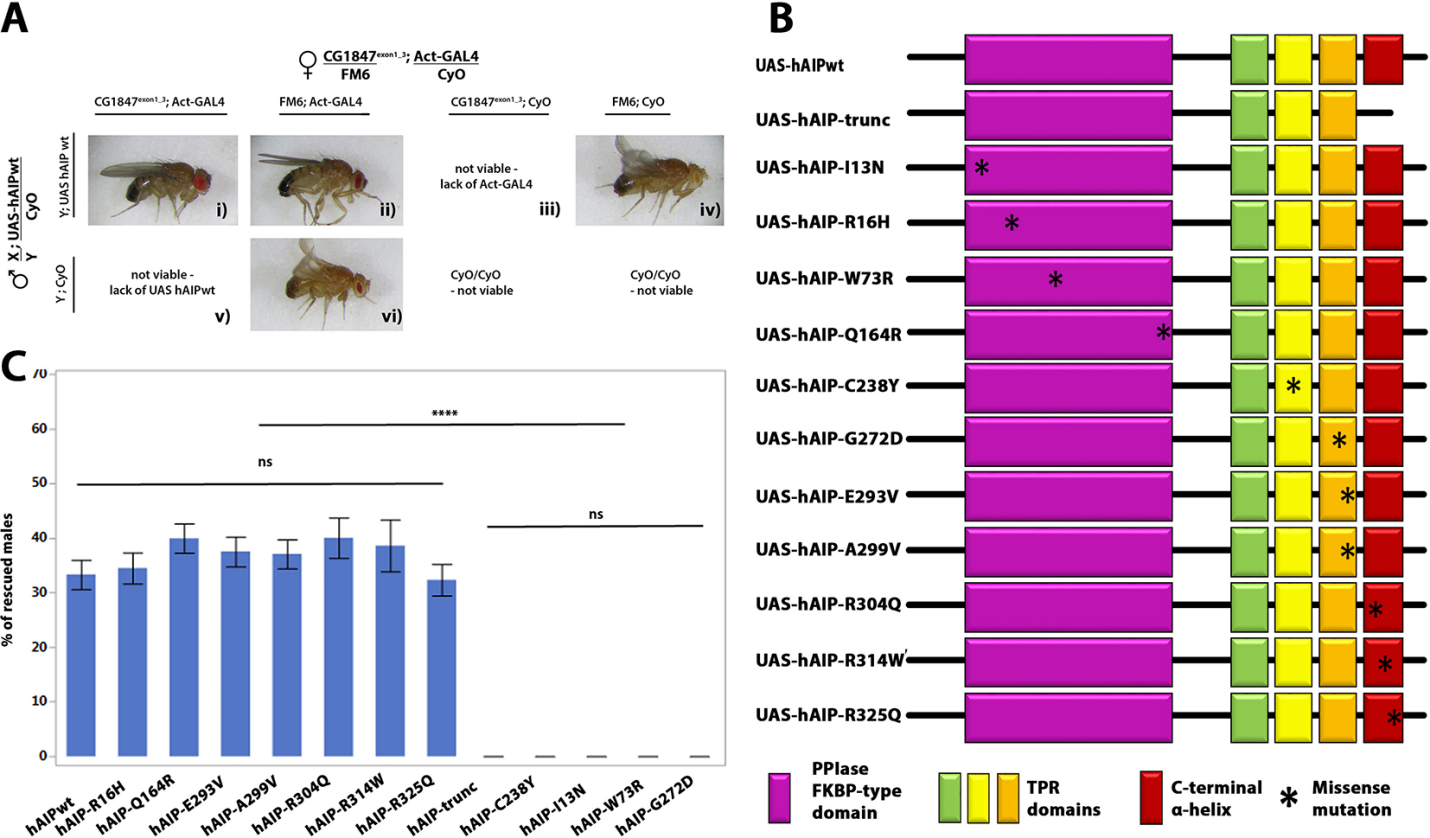
Human *AIP* functionally complements the *Drosophila* orthologue. (A) Transgenic males with h*AIP* rescue construct were crossed to heterozygous *CG1847* deficient females. The ubiquitous *actin-Gal4* driver was used to drive the expression of the *UAS-hAIP* constructs during fly development. Panel (**i**) Images of F1 viable rescued males. Males expressing wt h*AIP* in the *CG1847* mutant background (mutant *CG1847* allele inherited from their maternal X chromosome and expression of the h*AIP* transgene on chromosome 2); panels (iii) and (v): males inheriting the mutant *CG1847* allele and lacking hAIP expression are not viable. These two genotypes also serve as internal negative controls. Panels (ii) and (iv): males lacking the *CG1847* mutation are viable. (B) Schematic diagram of *UAS-hAIP* constructs. Top, wt UAS-h*AIP*. Second line: artificially generated truncated hAIP lacking the seventh alpha helix. Transgenic lines for 11 different h*AIP* missense mutations (p.R16H, p.C238Y, p.A299V, p.R304Q, p.I13N, p.W73R, p.Q164R, p.G272D, p.E293V, p.R314W and p.R325Q) were also generated. The approximate position of amino acid changes introduced are indicated with an asterisk. Protein domains are indicated by the colour code shown below the deletion construct assembly (the colours of the domains match what is shown in the 3D model, Figure 1). (C) Quantitative analysis of *in vivo* rescue experiments using h*AIP* missense variants (n=6). Successful rescue of lethality was scored as the presence of males with the genotype *CG1847^exon1_3^/Y; actin-Gal4/UAS-hAIP*, which lacks endogenous *CG1847*. Error bars represent SE of the mean. Significant differences are indicated by asterisks (****P<0.0001). AIP, aryl hydrocarbon receptor-interacting protein gene; TPR, tetratricopeptide repeat; wt, wild type.

Since *CG1847*-deficient males die at an early larval stage, they could serve as a useful model to test the functional conservation between human and *Drosophila AIP*. We used the *UAS-GAL4* system to express a human *AIP* (UAS-h*AIP*) transgene under the control of a ubiquitously active promoter (*actin-Gal4*) and assessed its ability to rescue the lethality of *CG1847* mutant males. Ubiquitous expression of UAS-h*AIP-*wt was able to functionally replace *CG1847* and rescue the lethality of *Drosophila CG1847^exon1_3^* mutants (Figure 3A), confirming that *AIP* is functionally conserved between flies and humans. Moreover, the proportion of viable F1 males carrying the rescue h*AIP* construct was close to the expected proportion (33% observed vs 25% expected in case of full rescue, corresponding to 1 of 4 viable genotypes). Flies expressing the UAS-h*AIP-*wt construct could therefore be used as a positive control for testing h*AIP* variants with unknown significance identified in patients with pituitary adenomas. We also generated transgenic flies with a h*AIP* construct containing a truncation mutation of the last α-helix of the AIP protein—known to be crucial for AIP function^26 34^—and used this as a positive (pathogenic) control representing a non-functional AIP protein (Figure 3B). This truncated h*AIP* was unable to rescue the *CG1847* ^exon1_3^ mutant as no viable males were found expressing the truncated h*AIP* variant (Figure 3C).

### Human *AIP* missense variants differ in their ability to rescue *CG1847* insufficiency

Having demonstrated that wt h*AIP* expression in *CG1847* knockout flies is able to rescue male lethality, while a truncated version is not, we next tested the rescue ability of 11 h*AIP* missense variants (p.I13N, p.R16H, p.W73R, p.Q164R, p.C238Y, p.G272D, p.E293V, p.A299V, p.R304Q, p.R314W and p.R325Q), found as germline variants in patients with pituitary adenomas (Figure 3B).

Out of all these tested variants, we selected one known to be a relatively common polymorphism (p.R16H) as a negative control, and two variants, a truncation mutation and the p.C238Y missense pathogenic mutation, as positive controls. There are considerable amount of data showing that p.R16H is a benign variant; it does not segregate with the disease^35^ and various in silico predictions and functional studies (online supplementary material 2) suggest that it is a benign polymorphism.^36^ Truncating variants are known to be pathogenic, while the p.C238Y missense variant is also known to be pathogenic, based on segregation, in silico testing, *in vitro* functional studies (cell proliferation^13^ and the PDE4A5 binding assays^16^) and half-life data^4^ (online supplementary material 2).

Given that all transgenes were site-specifically integrated into the genome, they are predicted to be expressed at similar levels. Clinical and genetic data available for these missense variants are presented in online supplementary material 2.

For each missense variant, we compared the proportion of rescued males with the corresponding proportion obtained with the wt and truncated h*AIP* controls (Figure 3C). h*AIP* variants separated into two groups: the p.R16H, p.Q164R, p.E293V, p.A299V, p.R304Q, p.R314W and p.R325Q variants rescued the lethal *CG1847^exon1_3^* phenotype at a similar rate as wt h*AIP* (Figure 3C). In contrast, four missense variants (p.C238Y, p.I13N, p.W73R and p.G272D) were unable to rescue the male lethality of *CG1847* mutants (P=0.0001) similar to the truncated *AIP* variant. The data obtained using our *in vivo* model supports and strengthens the clinical and bioinformatics data indicating that p.C238Y, p.I13N, p.W73R and p.G272D *AIP* variants are pathogenic. However, our findings suggest that the p.R16H, p.Q164R, p.E293V, p.A299V, p.R304Q, p.R314W and p.R325Q variants are functionally normal sequence alterations in our experimental setting.

### h*AIP* rescue constructs have equivalent expression levels

**Figure 4:**
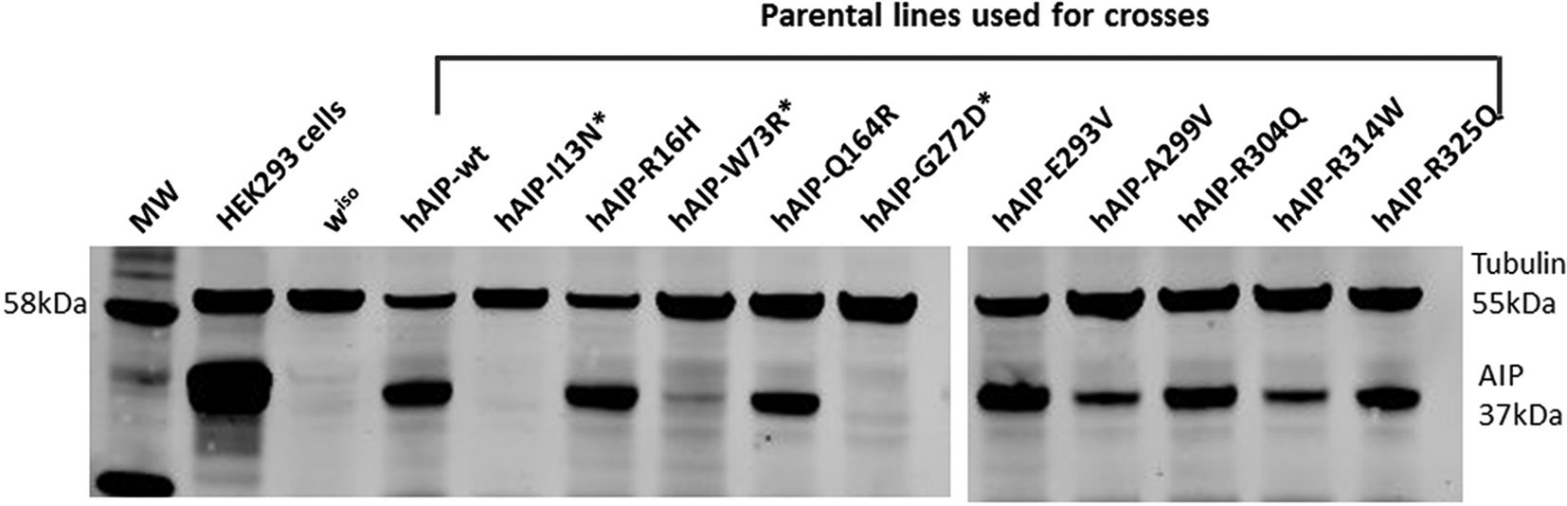
The different AIP constructs, wild-type human *AIP* (hAIP-wt) and various missense variants, show equivalent expression levels when ubiquitously expressed using the *actin-Gal4* driver. CG1487 protein expression was not detected in wild-type flies (w^iso^), suggesting the anti-AIP antibody is specific for the human protein and does not recognise endogenous CG1487. β-tubulin was used as loading. Surviving males I13N*, W73R* and G272D* are viable as they have endogenous CG1487 but no hAIP due to non-disjunction (further details of the non-disjunction phenomenon is presented in online supplementary material 1). AIP, aryl hydrocarbon receptor-interacting protein gene.

As ubiquitous expression of wt, p.R16H, p.Q164R, p.E293V, p.A299V, p.R304Q, p.R314W and p.R325Q h*AIP* resulted in rescue of *CG1847^exon1_3^* mutant males, total protein was extracted from fly heads to perform western blot analysis. Analysis of hAIP protein in fly lysates revealed a 37 kDa band, equivalent to the band detected in human HEK293T cells used as positive control (figure 4). The various hAIP constructs show similar protein expression levels (figure 4). Normal flies (w^iso^) were used as a negative control to confirm that the AIP antibody we used does not detect the endogenous CG1847 protein. In addition, transgenic males carrying the UAS-h*AIP* constructs only, without a Gal4 driver, did not display a hAIP band, excluding any detectable ‘leakiness’ of the transgenic UAS constructs (online supplementary figure 5).

While the p.R16H, p.Q164R, p.E293V, p.A299V, p.R304Q, p.R314W and p.R325Q h*AIP* missense constructs were able to rescue male lethality and displayed robust AIP expression (figure 4) similar to the level of wt h*AIP*, lysates from non-disjunction males (resulting from crosses with females carrying the p.I13N, p.W73R or p.G272D transgenes, figure 4, marked as *) did not contain human AIP protein. This confirmed that these males are indeed resulted from a non-disjunction event and are viable due to a normal copy of *CG1847* (online supplementary figure 4).

## Discussion

Patients with loss-of-function *AIP* mutations suffer from pituitary adenomas. For missense variants, the assessment of the functional and physiological consequences is hampered by the lack of robust *in vitro* and *in vivo* assays. In the era of increasing sequence data on human subjects, one of the most challenging issues is distinguishing pathogenic from non-pathogenic variants. In this study, combining a knockout for *CG1847* with the Gal4/UAS binary expression system, we developed and optimised an *in vivo* system to ‘bioassay’ the pathogenicity of *AIP* variants found in patients with pituitary tumours.^37^

We have found that deletion of the *CG1847* results in lethality, similarly to *AIP* knockout mouse models,^28 29^ and re-introduction of *CG1847* rescued this phenotype, demonstrating the specificity of our KO model. We then exploited the experimental power of *Drosophila* genetics to evaluate the degree of functional conservation between human *AIP* and its fly orthologue by testing whether *CG1847^exon1_3^* mutant flies could be rescued by human *AIP.*

A study of 287 human disease genes found a total of 178 (62%) genes having likely homologues in *Drosophila.* 38 Alignment of the human and fruit fly AIP proteins shows almost 40% identity, similar to the average level of protein identity between *Drosophila* and mammals.^39^

As the human gene was able to functionally compensate for the deletion of the *Drosophila* orthologue, our data support the evolutionary conservation of *AIP* gene function.

Furthermore, we have examined the effects of *AIP* variants in our *Drosophila* model in order to determine their pathogenicity. If a specific *AIP* variant rescues the lethality phenotype of *CG1847^exon1_3^* mutant flies, this strongly suggests that the variant does not cause a major disruption of AIP function, at least with regard to *Drosophila* development. Conversely, failure to rescue the lethality phenotype indicates the variant is likely to be non-functional and could account for the human disease.

We investigated 11 *AIP* missense mutations identified in patients with pituitary adenoma. Summary of these mutations are listed in table 1 and further detailed in online supplementary material, part 1. Our negative and positive controls confirmed the validity of the assay; p.R16H rescued the lethality to a level similar to wt *AIP,* while the truncated and p.C238Y variants failed to do so.

**Table 1:** Characterisation of *AIP* missense mutations identified in the various patients and investigated in this study

Variant	MAF in GnomAD	Typical phenotype (young, growth hormone (GH)-secreting adenoma)	LOH	Functional data	In silico prediction	*Drosophila* rescued
c.38T>A, p.I13N	8.25E-06	Young, GH-secreting adenoma	Yes	Not studied	Probably damaging	No
c.47G>A, p.R16H	0.001956	Familial cases, GH-secreting adenoma	No	Non-pathogenic	Probably damaging	Yes
c.217T>C, p.W73R	0	Young, GH-secreting adenoma	Not studied	Not studied	Probably damaging	No
c.491A>G, p.Q164R	0	Young, GH-secreting adenoma	Not studied	Not studied	Benign	Yes
c.713G>A, p.C238Y	8.42E-06	Familial cases, GH-secreting adenoma	Yes	Pathogenic	Probably damaging	No
c.815G>A, p.G272D	0	Elderly, GH-secreting adenoma	Not studied	Not studied	Probably damaging	No
c.878A>T, p.E293V	0	Elderly, GH-secreting adenoma	Not studied	Not studied	Benign	Yes
c.896C>T, p.A299V	4.00E-04	No	Not studied	Non-pathogenic	Benign	Yes
c.911G>A, p.R304Q	0.001458	Multiple cases	No	Non-pathogenic	Benign	Yes
c.940C>T, p.R314W	4.03E-05	Young, GH-secreting adenoma	Not studied	Not studied	Probably damaging	Yes
c.974G>A, p.R325Q	0.000058	Young, prolactin-secreting adenoma	Yes	Non-pathogenic	Benign	Yes

AIP, aryl hydrocarbon receptor-interacting protein gene; MAF, minor allele frequency.

The lethality of *CG1847^exon1_3^* mutants was rescued by 7 of the 11 tested h*AIP* missense variants (p.R16H, p.Q164R, p.E293V, p.A299V, p.304Q, p.R314W and p.R325Q), while four variants (p.C238Y, p.I13N, p.W73R and p.G272D) failed to do so. These data suggest that the latter four variants have a significant functional impairment or are unstable^4^ and therefore could represent pathogenic variants (see further discussion on these variants in online supplementary material 2). A similar strategy has previously been used to understand the conservation, functional role or importance of specific protein domains in human and *Drosophila* orthologues^40–43^ and could be employed to support clinical decision making.

Determining whether a variant is a disease-causing one is a significant challenge in the management of patients carrying a missense *AIP* variant,^8^ or indeed in any other partially penetrant disease.^36^ Evaluation of variant segregation with the phenotype in large pedigrees is the initial approach for investigating rare mutations.^10^ However, in case of *AIP* variants, this method is less practical due to the incomplete penetrance of the disease and the rarity of large families. Currently, there is no single method that can invariably predict the correct American College of Medical Genetics and Genomics (ACMG) category (‘pathogenic’, ‘likely pathogenic’, ‘uncertain significance’, ‘likely benign’ and ‘benign’) for missense variants. Although in silico prediction pipelines are often used, their results are not reliable, as shown recently, for endocrine genes including *AIP*.^36^ In addition to clinical data, such as segregation and variant frequency in the general population, various *in vitro* and *in vivo* methods could be used to help in the decision making. Inevitably, all methods have pitfalls. *In vitro* studies may not accurately recreate the environment present in a living organism. In the case of missense mutations, the change in amino acid sequence could disrupt their tertiary structure, with consequences for folding, stability and availability of protein–protein interaction sites. Robust and repeatable functional studies performed in clinical laboratories, however, have a significant role according to the ACMG guidelines.^44^

It was previously shown that the intact amino acid sequence of the TPR domains of AIP is essential for a proper interaction among amino acid residues in neighbouring alpha helices^5^; if these amino acids are changed by missense mutations, the resulted misfolded proteins are usually unstable.^6^ Various *in vitro* studies were employed to evaluate *AIP* variants, such as LOH analysis, splicing assays, cell proliferation, PDE4A5 binding and protein turnover.^4 13 16 45^ As we do not fully understand the mechanism of tumourigenesis induced by lack of AIP, the assay we might use in *in vitro* studies may not represent the true function of AIP. We demonstrated that AIP is an essential gene and several functional and comparative genomic analyses confirmed that essential genes are conserved during evolution.^46–49^ The fact that AIP is highly conserved among the species supports the fact that it is a disease-associated protein.^50^ As signalling pathways involved in organ development, cell proliferation, cell survival and cell migration are highly conserved in *D. melanogaster*,^51 52^ the results of fruit fly studies were shown to be transferable to humans; more than half of the known human disease genes, have homologues in fruit fly.^53^ We employed a *Drosophila* model organism to discover the conserved role of AIP and to avoid potential confounding factors arising from the redundancy and variability that can be generated by the analysis of more complex organisms. However, *in vivo* experiments, such as our *Drosophila* bioassay, may not correctly predict the functionality of a variant. Mouse studies, although closer to humans than *Drosophila*, can also provide misleading conclusions in some diseases.^54^ However, the fact that we used the human AIP protein in our studies and that this was sufficient to rescue the developmental function of the *Drosophila* orthologue is potentially a major advantage of our *in vivo* system, and this could be an additional approach to help clinicians reach the right conclusion.

In summary, we have engineered an *in vivo* bioassay for characterising patient-based *AIP* variants. The data presented support the evolutionary conservation of the *AIP* gene. Deletions of the endogenous *Drosophila* orthologue resulted in lethality of the flies, while the human gene can compensate for this loss. Rescue patterns of missense *AIP* variants can complement clinical and bioinformatics data and inform clinical decision making regarding *AIP* variants.^8 16^ The benefit of cascade genetic screening and clinical follow-up has already been established in *AIP* mutation-positive families,^3 55 56^ while family members in kindreds with non-pathogenic variants could be spared the psychological and financial burden of genetic testing and clinical follow-up.
