# Supplementary material for: In vivo bioassay to test the pathogenicity of missense human AIP variants
Source: J Med Genet. 2018 Apr 9;55(8):522–9. doi: 10.1136/jmedgenet-2017-105191 (PMC6073908; doi:10.1136/jmedgenet-2017-105191)
Supplement: Supplementary file 1 [file jmedgenet-2017-105191supp001.docx]

# **Supplementary Material 1 –non-disjunction phenomenon**

The non-disjunction phenomenon and its effect upon sex-linked inheritance was identified and characterised by Bridges in 1913.^1-3^ Non-disjunction is defined as “an improper segregation of chromosomes after meiosis, due to a failure to separate sister chromatids in meiosis 1”. In *Drosophila melanogaster* the non-disjunction phenomenon takes place mainly in females. It has been established that if inter-chromosomal genetic material exchange (genetic recombination) is absent, as in cases of balancer chromosomes (used in our setup), normal meiotic chromosome segregation is perturbed and the frequency of non-disjunction is increased.^4^ In our case, non-disjunction occurs between the mutated X chromosome CG1847^exon1_3^ and the balancer X chromosome FM6 (primary non-disjunction). Furthermore, non-disjunction is even more frequent in XXY females (note: unlike in humans, in *Drosophila* XXY results in fertile females) than it is in normal XX females. The XXY segregation event is called ‘‘secondary non-disjunction”.^2 4^ In XXY females, the association of the two X chromosomes with the Y chromosome is maintained at a significantly higher rate when the cross-over is suppressed by the presence of a balancer chromosome. This was demonstrated in females harbouring an *FM7* balancer X chromosome (genotype *FM7/X/Y).* A twenty-fold higher frequency of X non-disjunction was reported in *FM7/X/Y* females (70.8%) compared with the frequencies of non-disjunction in XXY females (around 3%).^5^

In our *CG1847* deletion model the *FM6* balancer X chromosome was introduced to the mutant stock. Consequently, the rate of non-disjunction was inevitably significantly increased. During normal gamete formation, the alleles for each gene segregate during meiosis and will later combine following the Mendelian laws of inheritance (Supplementary Figure 1).

| ***CG1847^exon1_3^/ FM6*** | |  |  |
| --- | --- | --- | --- |
|  |  | ***CG1847^exon1_3^*** | ***FM6*** |
| **X/Y** | **Y** | *CG1847^exon1_3^/Y* | *FM6/Y* |
|  | **X** | *CG1847^exon1_3^/X* | *FM6/X* |

Supplementary Figure 1: Overview of normal meiosis in the *CG1847^exon1_3^* mutant stock.

In bold: The segregation of maternal and paternal alleles during normal meiosis results in four haploid gametes, each containing one set of chromosomes. Normal segregation of a nonrecombinant chromosome pair results in normal disjunction.

Out of the four possible chromosome combinations depicted in Supplementary Figure 1 the non-disjunction phenotype occurs at highest rate in *FM6 /X* females.

In the primary type of non-disjunction normal *CG1847^exon1_3^* and *FM6* female chromosomes fail to segregate from each other during meiosis and consequently both will be found in the egg. The combination of such *CG1847^exon1_3^/ FM6* and zero eggs with the X and the Y male chromosomes will result in four new types of zygotes, as shown in Supplementary Figure 2.

| ***CG1847^exon1_3^/ FM6*** | |  |  |
| --- | --- | --- | --- |
|  |  | ***CG1847^exon1_3^/FM6*** | **0** |
| **X/Y** | **Y** | *CG1847^exon1_3^/FM6/Y*  *Viable and fertile females* | *0 /Y*  *Not viable* |
|  | **X** | *CG1847^exon1_3^/FM6/X*  *Not viable* | *0 /X*  *Sterile males* |

Supplementary Figure 2: Primary non-disjunction in *CG1847^exon1_3^* mutant stock.

A Punnett square for segregation and recombination of *CG1847^exon1_3^* and *FM6* chromosomes through primary non-disjunction, and the possible resulting progeny.

The *CG1847^exon1_3^/FM6/Y* females (note: these animals are fertile females despite carrying a Y chromosome) are the result of the primary non-disjunction. These females remain in the stock as they are not phenotypically different than original stock *CG1847^exon1_3^/FM6.*

In addition, Bridges noticed that almost all cases of X non-disjunction in XXY females is due to XXY segregation.^2^ This particular type of non-disjunction is well described in *Drosophil*a^2 4 6 7^; such non-disjunctional events might result in females who inherit both X chromosomes from their mothers, while no sex chromosome comes from their fathers.

| ***CG1847^exon1_3^ /FM6/Y*** | |  |  |
| --- | --- | --- | --- |
|  |  | ***CG1847^exon1_3^ / FM6*** | ***Y*** |
| **X/Y** | **Y** | *CG1847^exon1_3^ / FM6 /*  *Y* | *Y/Y*  *Not viable* |
|  | **X** | *CG1847^exon1_3^ / FM6 /*  *X(not viable)* | *X/Y*  *Fertile males mimicking the rescue phenotype (they inherited an intact normal X chromosome from the male parent, while the Y chromosome is inherited from the female parent having undergone primary non-disjunction)* |

Supplementary Figure 3: Secondary non-disjunction in the female.

A Punnett square for segregation and recombination of *CG1847^exon1_3^* and *FM6* chromosomes through secondary non-disjunction, and the possible resulting progeny.

In our *in vivo* assay non-disjunction was demonstrated by developing a PCR-based genotyping system and by using statistical analysis to determine the significance of the results.

To calculate rescue efficiency of males and to confirm the genotypes of the F1 offspring (whether the viable males are rescued or XO), genomic DNA was extracted from a proportion of the rescued males from each rescue cross. For each male, three different PCR reactions were performed to determine their genotype: (1) detection of the mutant *CG1847^exon1_3^* allele; (2) detection of the *hAIP* transgene; and (3) amplification of the *PPr-Y* gene located on the Y chromosome, to detect the presence of the Y chromosome in animals that were phenotypically males. This is necessary because a wild type male phenotype can also be produced by offspring with an X0 genotype, which can occur by primary non-disjunction of the X chromosome pair in the mother (as presented above).

Supplementary Figure 4: Validation of rescue experiments by PCR genotyping.

**A) Representative male progeny obtained in rescue crosses**. i), iii) and v) correspond to Figure 3A). Image i) shows a rescued male of the expected genotype and phenotype. Images iii) and v) are the result of non-disjunction - males that are phenotypically similar to genotypes *CG1847^exon1_3^ /Y; UAS-hAIP/CyO* (genotype “iii)” in and *CG1847^exon1_3^ /Y; actin-Gal4/CyO* (genotype “v)” in Figure 3A). They do not carry the FM6 balancer chromosome (see the normal round shape of the eyes as they are missing the Bar allele). As these males have the *CyO* balancer chromosome inherited on the chromosome 2; consequently they are missing one component of the UAS-GAL4 system: either *UAS-hAIP* (iii) or the *actin-GAL4* (v), therefore could not express hAIP. **B) Genotyping males potentially rescued with the h*AIP-*wt construct.** The resulting males (Figure 3A panel i) were genotyped by PCR. Eight out of 12 males had the expected rescue genotype as they carried the *CG1847^exon1_3^* allele (1000 bp amplicon) and h*AIP* cDNA on chromosome 2. Males 5, 6, and 12 did not carry the mutant *CG1847* allele (top panel, 2500 bp amplicon), and resulted from maternal chromosome X non-disjunction. Although phenotypically males, flies 6 and 12 do not carry the Y chromosome. Note that for sample m9 the *CG1847* amplicon could not be amplified, possibly due to a technical issue. **C) Genotyping males resulting from primary and secondary non-disjunction:** The males presented in A) panel iii) and panel v) were also genotyped using PCR. All 5 males carried the wt *CG1847* allele (top panel, 2500 bp amplicon). In the first two males (expected genotype *CG1847^exon1_3^ /Y; UAS-hAIP/CyO*) a copy of hAIP cDNA was detected (inherited from the father). As expected, the h*AIP* copy was not detected in the other three males for which the expected genotype, based on their phenotype, is *CG1847^exon1_3^ /Y; actin-Gal4/CyO*. In addition, in some of these phenotypical males the Y chromosome was not detected (XO males). **D) Image of non-disjunction males (non-FM6 non-CyO males) resulting from crosses with the hAIPtrunc construct.** When the truncated version of hAIP construct was used in the rescue experiments a limited number of males with the expected phenotype (non-FM6 non-CyO) were found in the F1 generation. **E) Genotyping of non-*FM6* non-*CyO* males resulting from crosses with the h*AIP*-trunc construct**. All 8 males carried the normal *CG1847* allele (top panel, 2500 bp amplicon) and therefore are the product of non-disjunction. This proofs that the truncated version of hAIP is not able to rescue and can therefore be used as positive control.

In the rescue experiment with hAIP wt construct we noticed 2 types of adult males in the F1 progeny that were phenotypically similar to genotypes *CG1847^exon1_3^ /Y; UAS-hAIP/CyO* and *CG1847^exon1_3^ /Y; actin-Gal4/CyO* (Supplementary Figure 4A panels iii) and v)). These males were not expected to be viable, since they do not have the proper combination of both GAL4 driver and UAS-hAIP transgene on chromosome 2. However, although these males are missing either the GAL4 driver or the UAS-hAIP, as revealed by the PCR (Supplemental Figure 4C) they are viable because they actually inherited the wild-type *CG1847* through the non-disjunction phenomenon described above. Consequently they have aberrant genotypes. Some of these flies were also negative for the Y chromosome, which supports that they result from primary non-disjunction. These phenotypes were found in low numbers (in average 4% of viable male offspring).

Together, these results suggest that the non-FM6 males are due to chromosome non-disjunction in parent females (as described above). These particular phenotypes, observed in low numbers in subsequent experiments, were considered aberrant genotypes and, therefore, were excluded from statistical analysis.

Regarding the individuals that are phenotypically identical to the rescued males found in the F1 generation of the rescue crosses for hAIPtrunc construct: these males were also the result of the non-disjunction phenomenon*.* The aberrant males in the rescue experiments with hAIPtrunc and hAIP C238Y constructs were used in the statistical analysis as “non-rescue” to evaluate the “rescued or not rescued result” based on a significant difference.

**Supplementary Material 2– clinical and bioinformatics analysis of the missense *AIP* variants**

**AIP p.I13N: (rs376913545, c.38T>A, chr11: 67250667)**

This variant was described in a patient with early onset gigantism.^8^ The patient experienced first symptoms at the age of 16, including sudden increase in height, visual problems, widening of his feet, bone and articular knees pain, excessive sweating, and the expansion of interdental spaces. MRI scan revealed an invasive macroadenoma. Tumour tissue analysis showed loss of normal allele and low intensity of AIP staining, in according with previous published data. Family history was negative and this is the only published case with this change. As no functional assay was available for determining whether this amino-acid change is a mutation or a rare polymorphism the authors classified this variant as pathogenic based on clinical data and *in silico* predictions using PolyPhen-2, which designated this amino-acid change as ‘probably damaging’ (0.999 score with 0.14 sensitivity and 0.99 specificity). This variant has a low MAF (minor allele frequency) in ExAC^9 10^ of 0.000008. Our *in vivo* experiments support the pathogenicity as this genetic variant was unable to rescue the lethality of *GC1847* deficient males.

**AIP p.R16H (rs145047094, c.47G>A, chr11: 67250676)**

Several reports have suggested the p.R16H variant is not pathogenic. It was first reported in two cousins with acromegaly,^11^ but its pathogenicity was later questioned^12 13^ and subsequent reports suggest it represents a rare SNP.^14^ A recent article reported a 3 generation Italian FIPA family with the R16H AIP mutation identified in 8 different individuals. However, as only 2 individuals were diagnosed with pituitary adenomas, this variant should be regarded as a rare polymorphism.^14^ Guaraldi *et al.*^12^ have suggested this variant does not segregate with the disease. Moreover, it does not affect the AIP-RET interaction.^15^ In support of a non-pathogenic role, the LOH status was investigated in pituitary tumorous tissues of two patients and the wild-type allele was present.^13 16^ Furthermore, this variant it degrades at similar speed as the wild-type protein.^17^ It has an ExAc database allele frequency of 0.0019, with one homozygote being identified. The recently-released gnomAD database^10^ reports a similar frequency with 2 homozygotes. Importantly, our *in vivo* analysis supports the current model that suggests p.R16H does not represent a pathogenic allele of AIP.

**AIP p.W73R (c.217T>C, chr11:672545594)**

This AIP germline variant was identified in a sporadic patient diagnosed with gigantism (1.98 m) and an invasive pituitary adenoma (20 mm)^18^ resistant to somatostatin analogue therapy. As the patient did not undergo surgery, there is no data regarding AIP staining or LOH analysis in tumour tissues. The substitution affects a conserved amino-acid in N-terminus. Although the majority of the functional studies have focused on the TPR domains of AIP, data regarding the structure of the N-terminus and its binding to HSP90 has provided possible pathophysiological pathways for N-terminal AIP mutations.^19 20^ *In silico* prediction using PolyPhen-2 characterised p.W73R as probably damaging (1.000 score with 0.00 sensitivity and 1.00 specificity). No data on *in vitro* testing available. This mutation is not present in the ExAC database. In our *in vivo* experiments this genetic variant did not resulted in rescue of lethality so our study support the fact that this is a pathogenic variant.

**AIP p.Q164R (c.491A>G, chr11: 67257531)**

This unpublished variant was identified by a Romanian group in a young acromegalic female which, when diagnosed at 34 years old, presented the following symptoms: enlarged extremities, headaches and amenorrhea. MRI scans revealed a large invasive sellar mass on the left cavernous sinus with suprasellar extension. Patient had an unsuccessful transsphenoidal surgery followed by octreotide treatment to which the adenoma was resistant. Currently the disease is controlled with pasireotide. Other family members were not affected. To date, there is no data regarding LOH or AIP immunostaining in tumour samples or *in vitro* functional analysis. *In silico* prediction using PolyPhen-2 suggested this is a benign variant (0.001 score with 0.99 sensitivity and 0.15 specificity). This mutation is not present in the ExAC database. In our *in vivo* model, AIP p.Q164R was able to compensate for loss of CG1847, which suggests this variant is non-pathogenic.

**AIP p.C238Y (rs267606569, c.713G>A, chr11:67257854)**

The p.C238Y variant was described in a Mexican family with 3 members diagnosed with acromegaly.^21 22^ All available data indicate that C238Y is a true disease-associated mutation. Functional studies have revealed that it has a reduced ability to block cellular proliferation and no interaction with PDE4A5 was detected.^21^ The LOH status was investigated in pituitary tumours of three Mexican patients and the wild-type allele could not be detected in any of the samples. Based on the crystal structure of AIP, this amino-acid is predicted to be involved in protein folding. In agreement with this, Morgan *et al*. ^23^ suggested that the C238Y mutation causes destabilisation of the packaging of α and β helices of the second TPR motif and induces protein misfolding and aggregation. This protein is very unstable, being rapidly degraded and having a very short half-life compared to the wild-type AIP.^24^ A single allele has been identified in a Latin population in the ExAC database. The gnomAD database reports an allele frequency of 0.000004. In our present study the p.C238Y missense variant was used as positive control and was unable to compensate for loss of *CG1847*, which also supports the pathogenic role.

**AIP p.G272D: (c.815G>A, chr11:67258268)**

This variant has been recently detected in a 50 year old female patient diagnosed with acromegaly at the age of 21.^25^ MRI scans identified a 3.5 cm invasive pituitary adenoma which proved to be very resistant to treatment as the patient underwent three surgical interventions, received conventional and gamma-knife radiotherapies and had SSA and dopamine agonist combination therapy. However, the hormonal control was achieved only after the use of pegvisomant. No other cases of pituitary adenoma were identified in her family, but the patient’s daughter was found to be a carrier of this genetic variant. The p.G272D mutation is not reported in dbSNP, EVS, ExAC or gnomAD databases and, consequently, there is no information regarding its allelic frequency. A similar G-to-D mutation at amino-acid 272 was first observed to be pathogenic in the TPR-containing CDC23 protein from S. cerevisiae^26^ and was shown to inhibit the progression through the G2/M transition of the cell cycle. Later, in 2009, this change was investigated in the AIP protein by Meyer *et al*.^27^ Their results revealed that this mutation disrupts interaction with known interacting partners as hsp90 and AhR, further confirming its pathogenic role. PolyPhen2 reports this variant as probably damaging (0.977 score with 0.76 sensitivity and 0.96 specificity). Our study suggests that this mutation is pathogenic and, therefore, carriers should be periodically screened for possible development of pituitary adenoma.

**AIP p.E293V: (c.878A>T, chr11:67258349) and AIP p.R314W (rs375740557, c.940C>T, chr11:67258367)**

The p.E293V is an unpublished AIP variant identified in Romania in a 51 year old female diagnosed with acromegaly. The patient had an extensive skull base mass, with bilateral invasion into cavernous sinuses and suprasellar region, and compression of the optic chiasm. Owing to tumor growth and visual field impairment, the patient underwent transsphenoidal surgery. The postoperative investigations showed a large remnant tumor and a second, transcranial, surgery was performed, followed by radiotherapy. The disease is currently controlled, both biochemically and regarding the tumour size, with somatostatin analogues. Interestingly, *AIP* mutation screening of blood DNA revealed a second heterozygous *AIP* variant in this patient, c.940C>T, p.R314W, also investigated in this study (see below). The tumour tissue was not investigated for LOH of intensity of AIP staining. This patient did not have any relatives with acromegaly or pituitary adenoma. *In silico* PolyPhen-2 prediction suggests that E293V is probably benign (0.004 score with 0.97 sensitivity and 0.59 specificity). This variant is not reported in the public databases.

The p.R314W variant was initially identified in a 30 year old giant patient^18^ and also in the patient mentioned above. The onset of disease for the first patient was during adolescence, with symptoms manifested at around the age of 18: enlarged extremities, headaches and visual field defect. MRI scan revealed an extensive 58mm macroadenoma invasive into the left cavernous sinus and suprasellar extension. Due to tumor size and significant optic chiasm syndrome the patient had transsphenoidal surgery as first therapeutic option. He remained symptomatic and underwent a second surgery which was a transcranial operation, followed by radiotherapy. His remnant adenoma was still secreting excess GH, and demonstrated resistance to dopamine agonists. Disease control was achieved only after treatment with pegvisomant. Tumour tissue analysis was not performed for normal allele loss or for the intensity of AIP staining. This patient had no family history of pituitary adenoma. No functional assay was performed to determine the pathogenicity of p.R314W. The gnomAd reports an allele frequency of 2.069e-5.

As two unrelated cases carrying the same genetic variant were reported, the authors classified this missense mutation as a disease causing one. This was supported by PolyPhen-2 *in silico* predictions, which suggest this amino-acid change is probably damaging to the protein structure (0.999 score with 0.14 sensitivity and 0.99 specificity). However, based on the fact that one patient carried both of the mutations and both were able to rescue *CG1847* loss in our *in vivo Drosophila* analysis, we propose that the two variants are not pathogenic.

**AIP p.A299V (rs148986773, c.896C>T, chr11:67258367)**

The A299V variant was first described in a Dutch patient with a GH-secreting pituitary adenoma.^13^ This variant was identified in a family where the pathogenic p.R304* mutation was also detected. Two unaffected patients were reported to carry both changes on separate alleles. As complete homozygote knockout mice are not viable, based on these human data, the A299V variant is unlikely to have a functional impact. There were two unaffected patients with A299V only, while another young female carried only A299V and was diagnosed with a microprolactinoma at the age of 30, most likely representing a phenocopy. There is no LOH data available. Functional studies for the A299V *AIP* variant have shown that this amino-acid change does not affect the AIP-RET interaction^15^ and *in vitro* causes a non-significant reduction in PDE4A5 binding.^28^ A299V variant is localised between the second and the third TPR domains, in a pocket essential for the interaction of AIP with HSP90. This AIP region is among the most conserved ones^23^ and it is important for proper protein folding. Misfolded structures could be unstable and result in rapid degradation of the protein.^29^ While previously attempts to purify A299V resulted in protein aggregation^23^, more recently data have shown that the A299V mutation has only a slightly shorter half-life compared to wt. The MAF is 0.0004 in ExAc and 0.0005567 in gnomAD. All the A299V clinical and *in vitro* data are in favour of a non-pathogenic role, suggesting it may be a rare polymorphism.^28 30^ This is also in accordance with our *in vivo* results, showing that p.A299V AIP variant does not have a pathogenic role.

**AIP p.R304Q (rs104894190, c.911G>A, chr11:67258382)**

R304Q is categorised as pathogenic mainly based on clinical data. Multiple groups have identified this change in patients with pituitary adenomas.^13 15 16 21 28 31-35^ 23 patients have been reported, with some coming from familial cases.^36^ The mutation is located in the C-terminal α-7 helix of the AIP protein, which is a CpG island hotspot. However, functional data do not support the pathogenicity as interaction with both PDE4A5^28^ and RET^15^ are not disrupted. Moreover, this variant degrades at similar speed as the wild-type protein.^17^ Interestingly, the ExAC database reports a MAF of 0.001458 and the presence of this variant in homozygosity in two Europeans. The gnomAD reports a MAF of 0.001532. We decided to test the p.R304Q variant in our *in vivo* studies based on the increasing discrepancy between the numerous clinical cases and functional studies. Although the clinical data is very convincing, our results do not support a pathogenic role for R304Q, as this *AIP* variant was able to rescue *Drosophila* lethality. Our data, in concert with the 2 homozygous cases reported in the ExAC and gnomAD databases, made us conclude that p.R304Q alone is not responsible for causing of pituitary adenomas. One possible explanation of the fact that this variant was identified in so many case is that p.R304Q may change the AIP activity in addition to a different gene (oligogenic model), which would explain the retention of the wild-type allele and no LOH in the pituitary adenoma tissue. One other possibility is that p.R304Q might be just a surrogate marker for another causative mutation with which it is associated by linkage of disequilibrium.

**AIP p.R325Q: (rs754619109, c.974G>A, chr11:67258445)**

This *AIP* missense mutation was identified in an 18 year old diagnosed with a PRL secreting adenoma. As it affects the last α-helix of AIP it was suggested to be a pathogenic variant. This was further supported by the identification of the same genetic variant in two members of the same family diagnosed with pituitary adenomas. Moreover, loss of the normal AIP allele was detected in LOH analysis of one of the patients. However, *in silico* analysis predicts this mutation is benign (0.001 score with 0.99 sensitivity and 0.15 specificity). In addition, no difference in protein stability was observed between the mutated and the wt versions of AIP.^17^ The ExAc/gnomAD MAF score is 0.000058. Our results are in agreement with a non-pathogenic role as the mutant AIP fully rescued the lethality of *CG1847* flies and the AIP protein was strongly expressed in the rescued males.

# **Supplementary Material 3 – the anti-AIP antibody is specific for human protein**


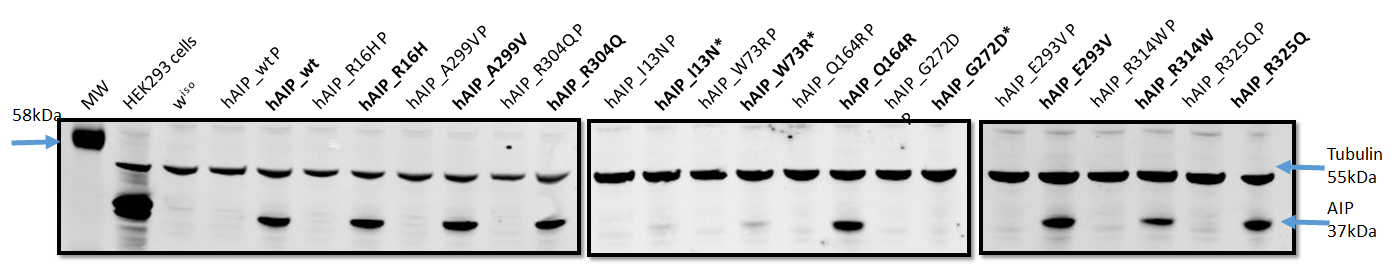


Supplementary Figure 5: The anti-AIP mouse antibody from NOVUS is specific for human protein.

The used antibody anti-AIP mouse (35-2) are specific for human protein as cannot detect the Drosophila protein. hAIP expression was not detected neither in wild-type flies (w^iso^) or in males carrying the UAS-hAIP transgenes in the absence of the *actin-Gal4* driver (P - Paternal generation). hAIP expression was only detected when *actin-Gal4* was combined with individual transgenic stocks (written in bold), suggesting that there is no residual expression of pUAS constructs. Surviving males marked as I13N*, W73R* and G272D* are viable as they have endogenous CG1487 but no hAIP due to non-disjunction.Immunoblot assays were performed with total protein extracts from adult male heads. β-tubulin was used as loading control.

Reference List

1. Bridges CB. Partial Sex-Linkage in the Pigeon. *Science* 1913;**37**(942):112-3.

2. Bridges CB. Non-Disjunction as Proof of the Chromosome Theory of Heredity. *Genetics* 1916;**1**(1):1-52.

3. Bridges CB. Non-Disjunction as Proof of the Chromosome Theory of Heredity (Concluded). *Genetics* 1916;**1**(2):107-63.

4. Cooper KW. A new theory of secondary non-disjunction in female Drosophila melanogaster. *Proc Natl Acad Sci U S A* 1948;**34**(5):179-87.

5. Xiang Y, Hawley RS. The mechanism of secondary nondisjunction in Drosophila melanogaster females. *Genetics* 2006;**174**(1):67-78.

6. Sturtevant AH, Beadle GW. The relations of inversions in the X chromosome of Drosophila Melanogaster to crossing over and disjunction. *Genetics* 1936;**21**(5):554-604.

7. O'Tousa J. Meiotic chromosome behavior influenced by mutation-altered disjunction in Drosophila melanogaster females. *Genetics* 1982;**102**(3):503-24.

8. Salvatori R, Daly AF, Quinones-Hinojosa A, Thiry A, Beckers A. A clinically novel AIP mutation in a patient with a very large, apparently sporadic somatotrope adenoma. *EDM Case Reports* 2014;**2014**:140048.

9. Exome Aggregation Consortium (ExAC). Secondary Exome Aggregation Consortium (ExAC). <http://exac.broadinstitute.org>

10. Lek M, Karczewski KJ, Minikel EV, Samocha KE, Banks E, Fennell T, O'Donnell-Luria AH, Ware JS, Hill AJ, Cummings BB, Tukiainen T, Birnbaum DP, Kosmicki JA, Duncan LE, Estrada K, Zhao F, Zou J, Pierce-Hoffman E, Berghout J, Cooper DN, Deflaux N, DePristo M, Do R, Flannick J, Fromer M, Gauthier L, Goldstein J, Gupta N, Howrigan D, Kiezun A, Kurki MI, Moonshine AL, Natarajan P, Orozco L, Peloso GM, Poplin R, Rivas MA, Ruano-Rubio V, Rose SA, Ruderfer DM, Shakir K, Stenson PD, Stevens C, Thomas BP, Tiao G, Tusie-Luna MT, Weisburd B, Won HH, Yu D, Altshuler DM, Ardissino D, Boehnke M, Danesh J, Donnelly S, Elosua R, Florez JC, Gabriel SB, Getz G, Glatt SJ, Hultman CM, Kathiresan S, Laakso M, McCarroll S, McCarthy MI, McGovern D, McPherson R, Neale BM, Palotie A, Purcell SM, Saleheen D, Scharf JM, Sklar P, Sullivan PF, Tuomilehto J, Tsuang MT, Watkins HC, Wilson JG, Daly MJ, MacArthur DG, Exome Aggregation C. Analysis of protein-coding genetic variation in 60,706 humans. *Nature* 2016;**536**(7616):285-91.

11. Daly AF, Vanbellinghen JF, Khoo SK, Jaffrain-Rea ML, Naves LA, Guitelman MA, Murat A, Emy P, Gimenez-Roqueplo AP, Tamburrano G, Raverot G, Barlier A, De Herder W, Penfornis A, Ciccarelli E, Estour B, Lecomte P, Gatta B, Chabre O, Sabate MI, Bertagna X, Garcia Basavilbaso N, Stalldecker G, Colao A, Ferolla P, Wemeau JL, Caron P, Sadoul JL, Oneto A, Archambeaud F, Calender A, Sinilnikova O, Montanana CF, Cavagnini F, Hana V, Solano A, Delettieres D, Luccio-Camelo DC, Basso A, Rohmer V, Brue T, Bours V, Teh BT, Beckers A. Aryl hydrocarbon receptor-interacting protein gene mutations in familial isolated pituitary adenomas: analysis in 73 families. *J Clin Endocr Metab* 2007;**92**(5):1891-6.

12. Guaraldi F, Salvatori R. Familial isolated pituitary adenomas: from genetics to therapy. *Clin Transl Sci* 2011;**4**(1):55-62.

13. Georgitsi M, Raitila A, Karhu A, Tuppurainen K, Makinen MJ, Vierimaa O, Paschke R, Saeger W, van der Luijt RB, Sane T, Robledo M, de ME, Weil RJ, Wasik A, Zielinski G, Lucewicz O, Lubinski J, Launonen V, Vahteristo P, Aaltonen LA. Molecular diagnosis of pituitary adenoma predisposition caused by aryl hydrocarbon receptor-interacting protein gene mutations. *Proc Natl Acad Sci USA* 2007;**104**(10):4101-05.

14. Zatelli MC, Torre ML, Rossi R, Ragonese M, Trimarchi F, degli Uberti E, Cannavo S. Should aip gene screening be recommended in family members of FIPA patients with R16H variant? *Pituitary* 2013;**16**(2):238-44.

15. Vargiolu M, Fusco D, Kurelac I, Dirnberger D, Baumeister R, Morra I, Melcarne A, Rimondini R, Romeo G, Bonora E. The tyrosine kinase receptor RET interacts in vivo with aryl hydrocarbon receptor-interacting protein to alter survivin availability. *J Clin Endocr Metab* 2009;**94**(7):2571-8.

16. Cazabat L, Libe R, Perlemoine K, Rene-Corail F, Burnichon N, Gimenez-Roqueplo AP, Dupasquier-Fediaevsky L, Bertagna X, Clauser E, Chanson P, Bertherat J, Raffin-Sanson ML. Germline inactivating mutations of the aryl hydrocarbon receptor-interacting protein gene in a large cohort of sporadic acromegaly: mutations are found in a subset of young patients with macroadenomas. *Eur J Endocrinol* 2007;**157**(1):1-8.

17. Hernandez-Ramirez LC, Martucci F, Morgan RM, Trivellin G, Tilley D, Ramos-Guajardo N, Iacovazzo D, D'Acquisto F, Prodromou C, Korbonits M. Rapid Proteasomal Degradation of Mutant Proteins Is the Primary Mechanism Leading to Tumorigenesis in Patients With Missense AIP Mutations. *J Clin Endocr Metab* 2016;**101**(8):3144-54.

18. Screening of AIP mutations in young Romanian patients with sporadic pituitary adenomas. *15th International Congress of Endocrinology*; 2012 2012; Florence, Italy.

19. Linnert M, Haupt K, Lin YJ, Kissing S, Paschke AK, Fischer G, Weiwad M, Lucke C. NMR assignments of the FKBP-type PPIase domain of the human aryl-hydrocarbon receptor-interacting protein (AIP). *Biomol NMR Assign* 2012;**6**(2):209-12.

20. Linnert M, Lin YJ, Manns A, Haupt K, Paschke AK, Fischer G, Weiwad M, Lucke C. The FKBP-type domain of the human aryl hydrocarbon receptor-interacting protein reveals an unusual Hsp90 interaction. *Biochemistry* 2013;**52**(12):2097-107.

21. Leontiou CA, Gueorguiev M, van der Spuy J, Quinton R, Lolli F, Hassan S, Chahal HS, Igreja SC, Jordan S, Rowe J, Stolbrink M, Christian HC, Wray J, Bishop-Bailey D, Berney DM, Wass JA, Popovic V, Ribeiro-Oliveira A, Jr., Gadelha MR, Monson JP, Akker SA, Davis JR, Clayton RN, Yoshimoto K, Iwata T, Matsuno A, Eguchi K, Musat M, Flanagan D, Peters G, Bolger GB, Chapple JP, Frohman LA, Grossman AB, Korbonits M. The role of the aryl hydrocarbon receptor-interacting protein gene in familial and sporadic pituitary adenomas. *J Clin Endocr Metab* 2008;**93**(6):2390-401.

22. Gadelha MR, Prezant TR, Une KN, Glick RP, Moskal SF, 2nd, Vaisman M, Melmed S, Kineman RD, Frohman LA. Loss of heterozygosity on chromosome 11q13 in two families with acromegaly/gigantism is independent of mutations of the multiple endocrine neoplasia type I gene. *J Clin Endocr Metab* 1999;**84**(1):249-56.

23. Morgan RML, Hernandez Ramirez LC, Trivellin G, Zhou L, Roe SM, Korbonits M, Prodromou C. Structure of the TPR domain of AIP: lack of client protein interaction with the C-terminal á-7 helix of the TPR domain of AIP is sufficient for pituitary adenoma predisposition. *PLoS One* 2012;**7**(12):e53339.

24. The enhanced proteasomal degradation of AIP mutant proteins is a mechanism for AIP deficiency in AIP mutation-associated pituitary adenomas. *The Endocrine Society Annual Meeting ENDO,* ; 2015; San Diego, USA.

25. Karaca Z, Taheri S, Tanriverdi F, Unluhizarci K, Kelestimur F. Prevalence of AIP mutations in a series of Turkish acromegalic patients: are synonymous AIP mutations relevant? *Pituitary* 2015;**18**(6):831-7.

26. Sikorski RS, Michaud WA, Hieter P. p62cdc23 of Saccharomyces cerevisiae: a nuclear tetratricopeptide repeat protein with two mutable domains. *Mol Cell Biol* 1993;**13**(2):1212-21.

27. Meyer BK, Petrulis JR, Perdew GH. Aryl hydrocarbon (Ah) receptor levels are selectively modulated by hsp90-associated immunophilin homolog XAP2. *Cell stress chaperon* 2000;**5**(3):243-54.

28. Igreja S, Chahal HS, King P, Bolger GB, Srirangalingam U, Guasti L, Chapple JP, Trivellin G, Gueorguiev M, Guegan K, Stals K, Khoo B, Kumar AV, Ellard S, Grossman AB, Korbonits M, International FC. Characterization of aryl hydrocarbon receptor interacting protein (AIP) mutations in familial isolated pituitary adenoma families. *Hum Mutat* 2010;**31**(8):950-60.

29. Goldberg AL. Protein degradation and protection against misfolded or damaged proteins. *Nature* 2003;**426**(6968):895-9.

30. Williams F, Hunter S, Bradley L, Chahal HS, Storr HL, Akker SA, Kumar AV, Orme SM, Evanson J, Abid N, Morrison PJ, Korbonits M, Atkinson AB. Clinical experience in the screening and management of a large kindred with familial isolated pituitary adenoma due to an aryl hydrocarbon receptor interacting protein (AIP) mutation. *J Clin Endocr Metab* 2014;**99**(4):1122-31.

31. Cazabat L, Bouligand J, Salenave S, Bernier M, Gaillard S, Parker F, Young J, Guiochon-Mantel A, Chanson P. Germline AIP mutations in apparently sporadic pituitary adenomas: prevalence in a prospective single-center cohort of 443 patients. *J Clin Endocr Metab* 2012;**97**(4):E663-70.

32. Tichomirowa MA, Barlier A, Daly AF, Jaffrain-Rea ML, Ronchi C, Yaneva M, Urban JD, Petrossians P, Elenkova A, Tabarin A, Desailloud R, Maiter D, Schurmeyer T, Cozzi R, Theodoropoulou M, Sievers C, Bernabeu I, Naves LA, Chabre O, Montanana CF, Hana V, Halaby G, Delemer B, Aizpun JI, Sonnet E, Longas AF, Hagelstein MT, Caron P, Stalla GK, Bours V, Zacharieva S, Spada A, Brue T, Beckers A. High prevalence of AIP gene mutations following focused screening in young patients with sporadic pituitary macroadenomas. *Eur J Endocrinol* 2011;**165**(4):509-15.

33. Cuny T, Pertuit M, Sahnoun-Fathallah M, Daly A, Occhi G, Odou MF, Tabarin A, Nunes ML, Delemer B, Rohmer V, Desailloud R, Kerlan V, Chabre O, Sadoul JL, Cogne M, Caron P, Cortet-Rudelli C, Lienhardt A, Raingeard I, Guedj AM, Brue T, Beckers A, Weryha G, Enjalbert A, Barlier A. Genetic analysis in young patients with sporadic pituitary macroadenomas: besides AIP don't forget MEN1 genetic analysis. *Eur J Endocrinol* 2013;**168**(4):533-41.

34. Occhi G, Jaffrain-Rea ML, Trivellin G, Albiger N, Ceccato F, De Menis E, Angelini M, Ferasin S, Beckers A, Mantero F, Scaroni C. The R304X mutation of the aryl hydrocarbon receptor interacting protein gene in familial isolated pituitary adenomas: Mutational hot-spot or founder effect? *J Endocrinol Invest* 2010;**33**(11):800-5.

35. Pardi E, Marcocci C, Borsari S, Saponaro F, Torregrossa L, Tancredi M, Raspini B, Basolo F, Cetani F. Aryl hydrocarbon receptor interacting protein (AIP) mutations occur rarely in sporadic parathyroid adenomas. *J Clin Endocr Metab* 2013;**98**(7):2800-10.

36. Hernandez-Ramirez LC, Gabrovska P, Denes J, Stals K, Trivellin G, Tilley D, Ferrau F, Evanson J, Ellard S, Grossman AB, Roncaroli F, Gadelha MR, Korbonits M, International FC. Landscape of Familial Isolated and Young-Onset Pituitary Adenomas: Prospective Diagnosis in AIP Mutation Carriers. *J Clin Endocr Metab* 2015;**100**(9):E1242-54.
